# Determination of the microscopic acid dissociation constant of piperacillin and identification of dissociated molecular forms

**DOI:** 10.3389/fchem.2023.1177128

**Published:** 2023-04-26

**Authors:** Guoao Li, Yaling Wang, Chengyi Sun, Fei Liu

**Affiliations:** ^1^ Beijing Key Laboratory of Water Resources and Environmental Engineering, School of water resource and Environment, China University of Geosciences (Beijing), Beijing, China; ^2^ Beijing Municipal Research Institute of Eco-Environmental Protection, Beijing, China; ^3^ National Engineering Research Center for Urban Environmental Pollution Control, Beijing, China

**Keywords:** acid dissociation constant, piperacillin, potentiometric titration, *β*-lactam antibiotics, molecular forms

## Abstract

For amphoteric *ß*-lactam antibiotics, the acid dissociation constant (p*K*_a_) is a fundamental parameter to characterize physicochemical and biochemical properties of antibiotics and to predict persistence and removal of drugs. p*K*_a_ of piperacillin (PIP) is determined by potentiometric titration with a glass electrode. Electrospray ionization mass spectrometry (ESI-MS) is creatively applied to verify the reasonable p*K*_a_ value at every dissociation step. Two microscopic p*K*_a_ values (3.37 ± 0.06 and 8.96 ± 0.10) are identified and attributed to the direct dissociation of the carboxylic acid functional group and one secondary amide group, respectively. Different from other *ß*-lactam antibiotics, PIP presents a dissociation pattern where direct dissociation is involved instead of protonation dissociation. Moreover, the degradation tendency of PIP in an alkaline solution may alter the dissociation pattern or dismiss the corresponding p*K*_a_ of the amphoteric *ß*-lactam antibiotics. This work offers a reliable determination of the acid dissociation constant of PIP and a clear interpretation of the effect of stability of antibiotics on the dissociation process.

## 1 Introduction

Piperacillin (PIP), which belongs to penicillin antibiotics containing the *ß*-lactam moiety, is widely and frequently used in medicinal and veterinary therapy (Hsia et al., 2019) to prevent post-operative infection complications (Milne and Waterworth, 1978; Pastena et al., 2020). A high daily dose of PIP (about 12–16 g) results in a high residue level in the patient’s blood and feces (Carlier et al., 2015). Moreover, like other antibiotics, a large amount of PIP may migrate into the environment as persistent or pseudo-persistent substances creating risks to the ecological environment and human health (Polianciuc et al., 2020). Actually, PIP has been detected in underground water, with the highest detected concentration of 571 ng L^-1^ (Szekeres et al., 2018), as well as in surface water (Da Le et al., 2021; Danner et al., 2019; O'Flaherty and Cummins, 2017; Anh et al., 2021; Wu et al., 2020; Soran et al., 2017; Adams et al., 2002), drinking water treatment plants (Mahmood, Al-Haideri, and Hassan, 2019), wastewater (Faleye et al., 2017), and so on. As is known, the acidity/alkalinity of an amphoteric pharmaceutic substance is among the most fundamental properties for drug action (Alekseev, 2010; Charifson and Walters, 2014). The acid dissociation constant (p*K*_a_) is a characteristic parameter representing ionization equilibrium and predicting molecular form variations with respect to pH (Demiralay et al., 2012). For that, since p*K*_a_ has an influence on solubility and lipophilicity, biological enrichment, and toxicity, p*K*_a_ is definitely critical to absorption, distribution, metabolism, and excretion involved in the fields of environmental chemistry, biological chemistry, pharmaceutical chemistry, and medicinal development (Nural et al., 2020). Therefore, it plays an important role in determining the acid dissociation constant for understanding the persistence and removal of PIP.

p*K*_a_ values of pharmaceuticals can be determined indirectly *via* potentiometric titration (Evagelou, Tsantili-Kakoulidou, and Koupparis, 2003; Ke et al., 2016), UV or fluorescent spectrophotometry (Evagelou, Tsantili-Kakoulidou, and Koupparis, 2003), chromatography (Jančić et al., 2007), and the coupling method. Potentiometric titration in aqueous solutions is a simple and effective method and considered to be the most precise method for the determination of equilibrium constants (Ke et al., 2016). No additional derivative procedures or special functional groups or knowledge of all binding partners and their stoichiometry are required (Guo et al., 2016; Budhadev et al., 2020; Liao et al., 2021). For the treatment of the titration curve, the Henderson–Hasselbalch equation is often used to calculate the pH of a buffer. Yet, there is difficulty for weak polyprotic acids due to the overlaps of multiple acid–base equilibriums and titration jumps in some pH ranges. In this case, piecewise linear regression is helpful, in which the independent variable is segmented according to its value, and the linear regression is performed separately on these segments (Ke et al., 2016). Additionally, mass spectrometry is scarcely applied for the detection of p*K*_a_ values. Mass spectrometry is used for resolving degradation/dissociation products (Ahmed et al., 2012; Liang et al., 2017) and noncovalent interactions (Kempen and Brodbelt, 2000; Hardouin and Lange, 2005; Zhang et al., 2006; Erba and Zenobi, 2011). A recent review reported its potential for the determination of dissociation constants and giving information about the specificity of noncovalent interactions (Schulte et al., 2023). Several reports have presented evidence when properly controlled experimental conditions are used, electrospray ionization mass spectrometry (ESI-MS) has demonstrated its use in the detection and study of weakly bound forms. Its data reflect solution-phase chemistry, meaning that one should be able to derive binding affinities quantitatively from such data (Mathur et al., 2007; Jecklin et al., 2008). Considering this, the ESI-MS method is potential for the identification of acid dissociation products.

So far, p*K*_a_ values of some antibiotics are determined, and ionizable moieties are analyzed via theory analysis and experimental validation (Montaudo, Caccamese, and Recca, 1975; Lin et al., 2004; Qiang and Adams, 2004; Andrasi et al., 2007; Babić et al., 2007; Kong et al., 2007; Rayer et al., 2014). Some studies apply p*K*_a_ to facilitate the exploration of the effect of pH on the removal of PIP by wastewater treatment technology (Mahmood, Al-Haideri, and Hassan, 2019), the mechanism of PIP decomposition (Xuexiang, 2014), and bioactive metabolic products of PIP and metabolic path. Recently, various novel methods have been developed to determine p*K*_a_ (Reijenga et al., 2013; Fuguet et al., 2015; Subirats et al., 2015), and p*K*_a_ values in multi-solvent systems are investigated (Sanli, Altun, and Alsancak, 2012; Eugene-Osoikhia, 2020). However, due to the complex chemical structure with diverse functional groups and multiple ionizable moieties in the PIP molecule, incomplete or scarce acid dissociation constants are published along with the dissociation patterns (Alekseev, 2010). Moreover, the prediction results are not always consistent with the actual situation (Ribeiro and Schmidt, 2017). A reliable determination of the acid dissociation constant of PIP and a clear interpretation are remained to be solved.

In this work, microscopic acid dissociation constants of PIP in an aqueous solution are determined by potentiometric titration with a glass electrode at a constant temperature. ESI-MS in the infusion mode (without LC) is used creatively to identify functional groups related to microcosmic acid dissociation constants during overlapping ionization processes. The distribution of various PIP dissociation forms versus solution pH is recognized.

## 2 Materials and methods

### 2.1 Reagents and solutions

PIP powder (CAS 61477-96-1, 97% purity) was purchased from Beijing Bionet Co., Ltd. The standard substance number is CB 0181853 with a molecular weight of 517.55. Solid sodium chloride (NaCl, AR grade) and hydrochloric acid (37 w% HCl, AR grade) were bought from Beijing Chemical Works Co., Ltd. Sodium hydroxide (NaOH, AR grade) was purchased from Sinopharm Chemical Reagent Co., Ltd. All chemicals were used as received without further purification.

PIP solutions: PIP stock solution (100 μmol L^-1^) was prepared by completely dissolving 0.103 g of the solid PIP powder into 2 L of ultrapure water. The concentration is lower than the solubility of PIP, 0.119 mg mL^-1^. Then, PIP stock solution was diluted using NaCl solution (0.1 mol L^-1^) to 5 μmol L^-1^, 10 μmol L^-1^, and 50 μmol L^-1^, respectively, with the same final total volume of 50.0 mL. Here, NaCl solution instead of ultrapure water was used as the electrolyte, contributing background ion strength to improve the sensitivity of potentiometric titration. As a blank control, the titration results of 0 μmol L^-1^ of PIP (that is, 0.1 mol L^-1^ of NaCl solution) were subtracted from titration volumes. All as-prepared PIP solutions were stored in 60-mL brown VOA bottles. Additionally, it was noted that only fresh PIP solutions can be used so that no photolysis, hydrolysis, or oxidative degradation occurs before potentiometric titration and mass spectrometry characterization.

Saturated NaOH solution was prepared and was diluted to about 0.2 mol L^-1^. This procedure can avoid the dissolution of carbonate in NaOH solution as much as possible. The prepared NaOH titrant was calibrated to be 0.2002 mol L^-1^. About 1.0 mol L^-1^ HCl was prepared by diluting the concentrated HCl reagent with ultrapure water, which was then calibrated to be about 1.0030 mol L^-1^.

Moreover, special attention should be paid to the following: 1) all ultrapure water used is purged by high-purity argon and boiled to remove carbon dioxide and oxygen. 2) The NaOH titrant is freshly prepared on the day of the experiment to prevent the absorption of carbon dioxide and any other chemicals from the ambient air. 3) All as-prepared solutions are uniformly stored at 25°C to minimize experimental errors.

### 2.2 Apparatus

Potentiometric titration is carried out using an automatic potentiometric titrator equipped with a pH glass electrode (877 Titrino plus, Metrohm, Switzerland). PIP solution and added titrant are mixed uniformly using a thermostatic magnetic stirrer (RCT Basic S 25, IKA, Germany). PIP is weighed using a 1/100,000 electronic scale (AB265-S, METTLER TOLEDO, Switzerland). Mass spectrometry characterization is performed on a high-performance liquid chromatography-triple quadrupole mass spectrometer (Xevo TQD, Waters, United States).

### 2.3 Potentiometric titration experiments

Potentiometric titration experiments of PIP solutions are carried out for PIP solution with different concentrations (5, 10, and 50 μmol L^-1^). First, HCl solution (1.0030 mol L^-1^) is titrated into PIP solution to initialize to pH 3.0 and kept steady for 10 min. Then, NaOH solution (0.2002 mol L^-1^) is continuously titrated into PIP solution to pH close to 11.0. The titration volume of each drop is set at 10 μL, and an interval of 10 s is set for pH equilibrium (fluctuation is no more than 0.01 pH). The solution system is kept in a water bath at 25 ± 1°C. Highly pure argon is purged continuously through the solution to avoid the contact of ambient air with the solution. pH variation versus titration volume of NaOH is automatically recorded. Since p*K*_a_ is independent of the concentration of the solution, for each type of dissociation site, p*K*_a_ values in triplicate for different concentrations are obtained.

### 2.4 Mass spectrometry experiments

About 500 μg mL^-1^ of PIP aqueous solution is prepared. The solution pH is manually tuned to 3.0, 5.0, 7.0, and 9.0, respectively, with HCl and NaOH solutions. Then, PIP solutions are qualitatively characterized on a high-performance liquid chromatography-triple quadrupole mass spectrometry in the infused injection mode.

## 3 Results and discussion

### 3.1 Microscopic p*K*_a_ (Micro-p*K*_a_)

**FIGURE 1:**
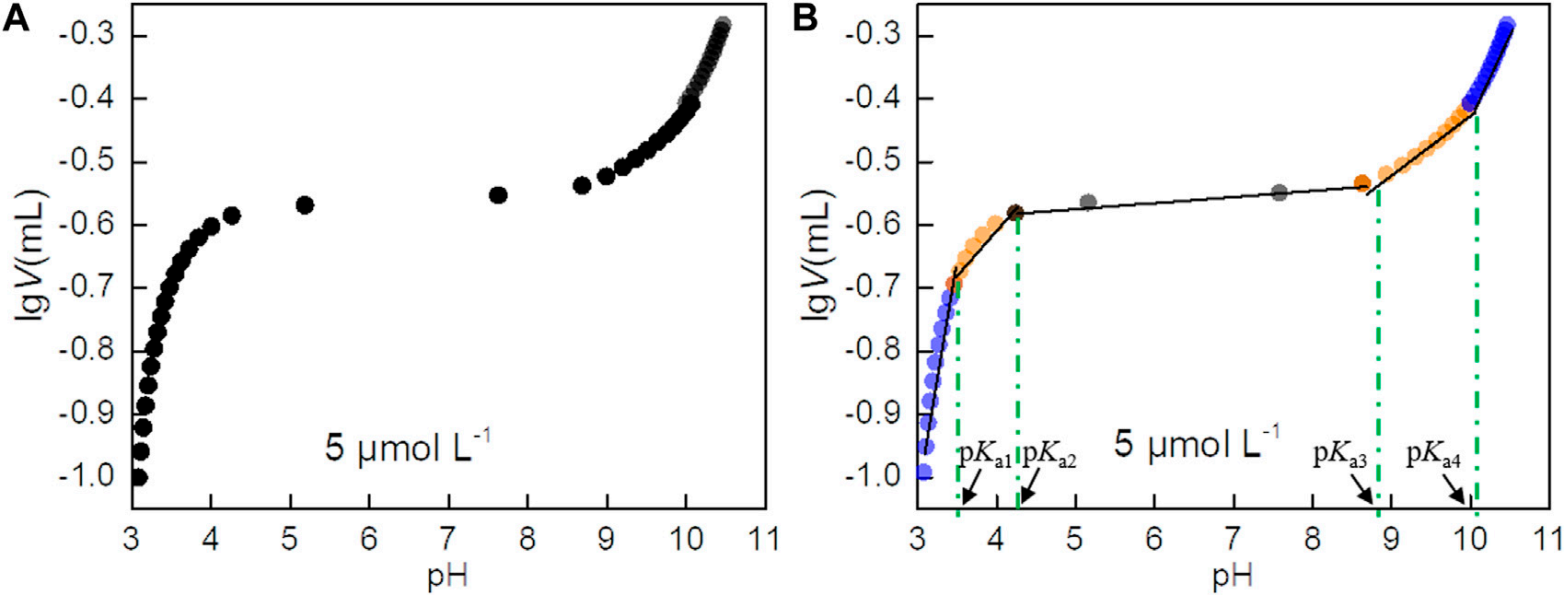
**(A)** Scatter plot of lgV versus pH. **(B)** Piecewise linear regression results of the lgV-pH data. Potentiometric titration is performed for 5, 10, and 50 μmol L-1 PIP solutions Here, Panel **A** is given for 5 μmol L^-1^ of PIP, and Supplementary Figure S1 and Supplementary Figure S2 are given for 10 and 50 μmol L^-1^ of PIP solutions.

All potential p*K*_a_ values of PIP are excavated through piecewise linear regression, following a method from the reference (Ke et al., 2016). Titration data in the form of lg*V*-pH are plotted, as shown in Figure 1A, and five linear fitting curves are presented (Figure 1B; Supplementary Table S1). The four potential p*K*_a_ values are indicated for generating at the connectors of two curves. Based on this method, the potential p*K*_a_ values of PIP at different concentrations are listed in Table 1. In addition, the average p*K*_a_ value at each step is obtained from PIP at different concentrations. The previous literature (Sörgel and Kinzig, 1993) reported that the piperacillin p*K*_a_ value was 4.14, and the determination method was not clear. Another piece of literature (Tsukinaka et al., 1982) reported that the p*K*_a_ value of piperacillin obtained by potentiometric titration was 2.9 at 35°C, with an ionic strength of 0.5. Our previous study measured that the p*K*_a_ value of piperacillin was 3.19 ± 0.02 at 25°C (published in Chinese). In these studies, only one p*K*_a_ was obtained, and there was no dissociation site information.

**TABLE 1:** Potential p*K*_a_ of PIP.

	5 μmol L^-1^	10 μmol L^-1^	50 μmol L^-1^	Average	SD	RSD %
p*K*_a1_	3.46	3.34	3.32	3.37	0.06	1.74
p*K*_a2_	4.18	4.07	3.95	4.07	0.09	2.32
p*K*_a3_	8.83	8.97	9.08	8.96	0.10	1.12
p*K*_a4_	10.04	9.96	9.99	9.99	0.03	0.32

**FIGURE 2:**
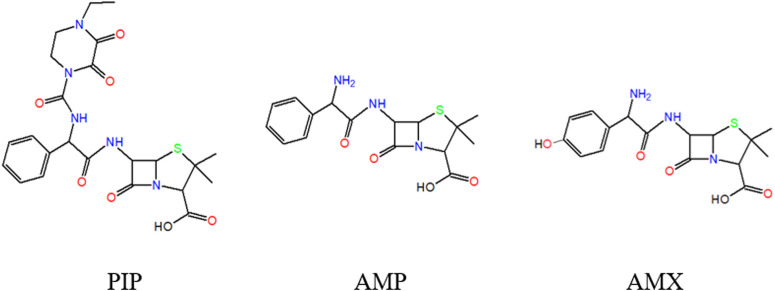
Schematic diagram of the molecular structure of PIP, AMP, and AMX.

The potential p*K*_a_ values of PIP can be explained by the molecule structure. According to the molecule structure of PIP (Figure 2), it is deduced that the value of p*K*_a2_ at about 4.07 is illogical and should be excluded referring to the literature about ampicillin (AMP) and amoxicillin (AMX) (Demiralay et al., 2012). Therefore, the other three values 3.37 ± 0.06, 8.96 ± 0.10, and 9.99 ± 0.03 are identified as potential p*K*_a_ values of PIP (Table 1).

### 3.2 Mass spectrometric characterization

**FIGURE 3:**
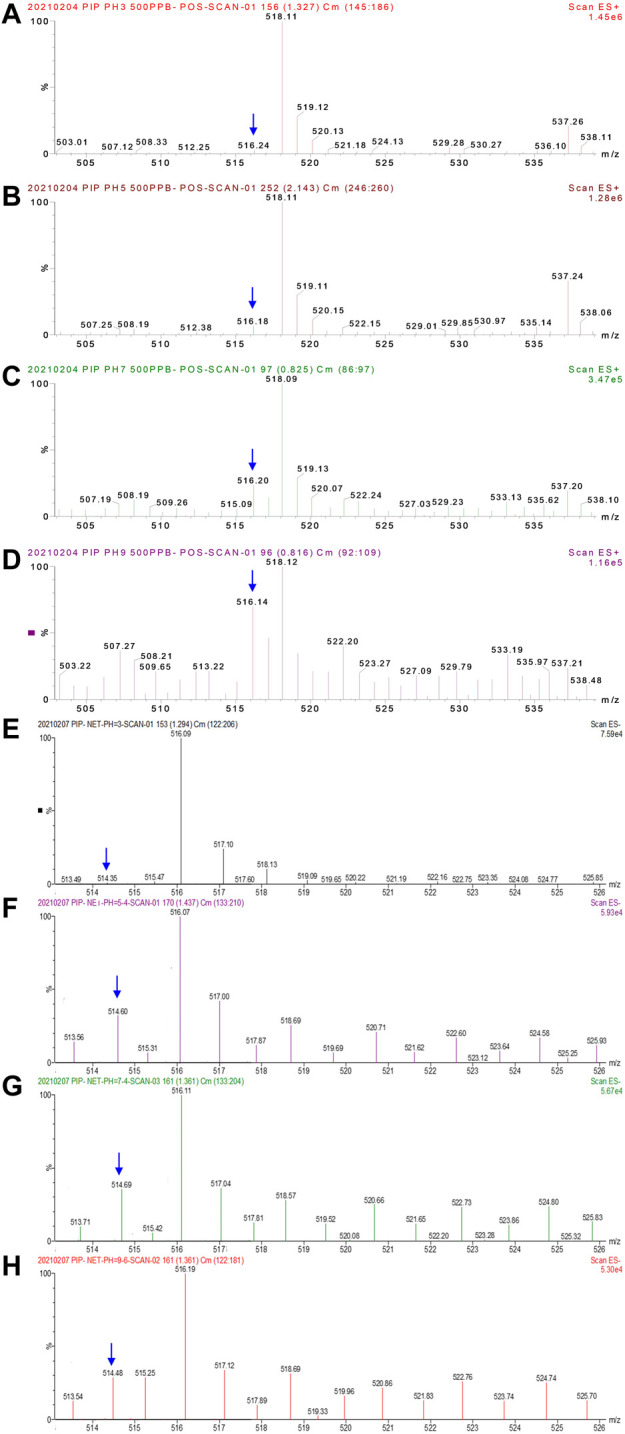
Mass spectrometry of PIP (intact molecular concentration of 500 μg mL^-1^) in positive **(A–D)** and negative **(E–H)** modes under different pH values. **(A)** pH 3.0, **(B)** pH 5.0, **(C)** pH 7.0, and **(D)** pH 9.0 in the positive mode; **(E)** pH 3.0, **(F)** pH 5.4, **(G)** pH 7.4, and **(H)** pH 9.6 in the negative mode. The full scale of signal intensity is presented as 2.15 × 10^6^,2.18 × 10^6^, 6.22 × 10^5^, 2.44 × 10^5^, 7.59 × 10^4^, 5.93 × 10^4^, 5.67 × 10^4^, and 5.30 × 10^4^. The difference in the molecular ion intensity demonstrates the occurrence of the dissociation or degradation of PIP.

Mass spectrometry of PIP in the positive ionization mode is shown in Figure 3 and Supplementary Figure S2. Since PIP has a molecular weight (M) of about 517, the mass charge ratio (m/z) at 518 is generally recognized as the characteristic molecular ion of PIP in the positive mode.

PIP does not tend to be protonated in an acid solution. As shown in Supplementary Figure S3 and Supplementary Figure S4, a mass spectrogram in the scanning mode (m/z 245–263 and m/z 150–184) demonstrates that there are no multiple-charged molecular ions, for example, [MH_n_]^n+^ (2 ≤ *n* ≤ 5), indicated by the absence of m/z at 259.5 and 173. This reveals that amide groups in PIP are not protonated in an acid solution.

**FIGURE 4:**
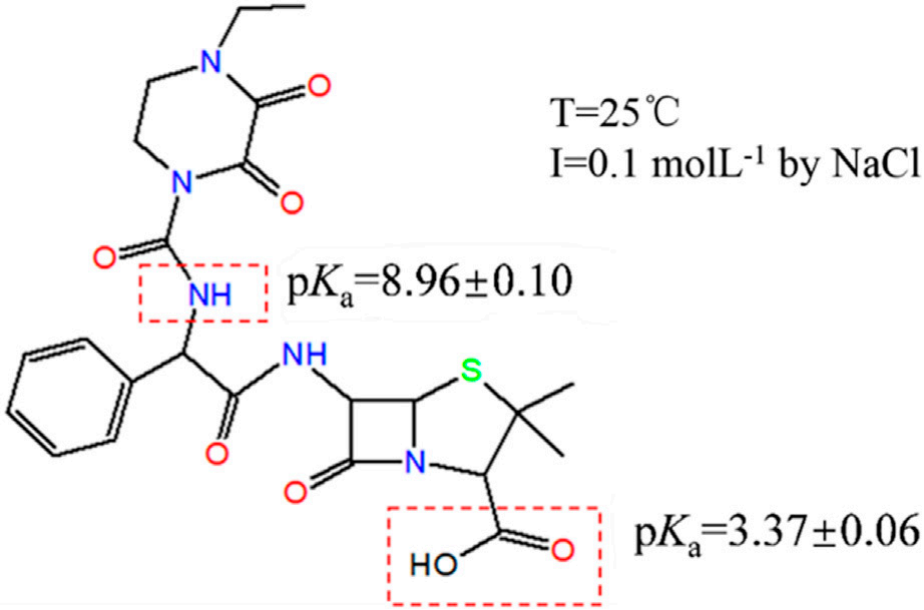
Relationship between p*K*_a_ of PIP and its structure.

Direct dissociation of amide groups in PIP is involved instead of protonation dissociation. Figure 3 presents the specific mass spectrometry of PIP (m/z 505–545). When the solution pH is tuned to 3.0 (Figure 3A), the predominant m/z is 518. The undissociated PIP molecule transforms to [MH]^+^ in positive electrospray, and then [MH]^+^ is detected. As the solution pH further increases to 5.0 (Figure 3B) and then changes to be neutral (pH 7.0 in Figure 3C) or alkaline (pH 9.0 in Figure 4D), the response at m/z 516 and 517 increases obviously along with the solution pH. That is to say, the content of substances with molecular weights M-2 and M-1 increases. It reveals that, in a neutral or alkaline solution, dissociation occurs in PIP via losing one or two H^+^ ions in advance, respectively. Dissociation happens to the carboxyl group and then possibly certain amide groups in PIP. Then, the two types of anions, [M–H]^‒^ and [M–2H]^2‒^, are immediately oxidized induced by the high potential on the capillary wall. Afterward, the oxidized products change to neutral pH (molecular weight M-1 and M-2) accompanied by the losing of electrons. Then, molecular ions [(M-1)]H^+^ and [(M-2)]H^+^ are detected. It reveals that amide groups in PIP directly dissociate one H^+^ ion instead of protonation dissociation.

### 3.3 Functional groups affordable for PIP micro-p*K*_a_

The affordable functional groups of PIP microscopic p*K*_a_ are parsed by structural analogy among PIP, AMP, and AMX (Figure 2). First, the single apparent acid dissociation constant (p*K*_a_ = 3.19 ± 0.02) is calculated by a direct method from the titration curve, demonstrating a feature of monoprotic acids. The micro-p*K*_a_ of PIP at 3.37 ± 0.06 is close to the apparent acid dissociation constant. Previous works report that the acid dissociation constant of carboxylic acid is at p*K*_a_ 2–4 (Martínez, 1989; Jaszczak and Kufelnicki, 2010a). p*K*_a_ values of AMP and AMX derived from the carboxylic group (Table 2) also consist of the aforementioned patterns. Therefore, it indicates that p*K*_a1_ of PIP (3.37 ± 0.06) is most probably attributed to the functional group of carboxylic acid.

**TABLE 2:** p*K*_a_ of AMP and AMX.

Antibiotic	p*K*_a1_	p*K*_a2_	p*K*_a3_	p*K*_a4_	Method	Reference
AMP	3.966	7.541	11.264		Spectrophotometric and reversed-phase liquid chromatography; calculation by Yasuda–Shedlovsky and mole fraction equations	Demiralay et al. (2012)
AMX	3.001	8.042	10.261	11.922		
AMP	2.66	7.10	11.34 (-CONH-)		Potentiometric titration and UV/VIS spectra	Jaszczak and Kufelnicki (2010a)
AMX	3.11	7.38	9.60 (-CONH-)			
AMP	2.66	7.24			Solubility experiment and calculation by a simplified perturbed hard sphere theory	Rudolph et al. (1999)
AMX	2.63	7.16				
AMP	2.14	7.37			Solubility experiment and calculation by a simplified perturbed hard sphere theory	Santana et al. (2010)
AMP	2.592	7.239			Potentiometric and spectrophotometric measurements; calculation by the Setschenow equation	Crea et al. (2012)
AMX	2.549	7.501	10.014			
AMX	2.41 (-COOH)	7.19 (-NH_3_^+^)	9.38 (-OH)		Potentiometric titrations and calculation by the MINIQUAD-75 program	Shoukry (1993)

Second, it is noted that PIP starts to break down in an alkaline situation. For example, PIP under a pH of 9.5 has degradation rate constants of 0.12 h^-1^ and 2.7 h^-1^ at 35°C in two paths (the content of intact PIP is 1) (Tsukinaka et al., 1982). Accordingly, the third p*K*_a4_ at 9.99 ± 0.03 should be excluded. Moreover, once the pH increases to 11, a complete degradation will immediately occur in hours (Tsukinaka et al., 1982; Mitchell et al., 2014). Considering this, no remaining functional groups will account for the additional p*K*_a_ >11. The degradation tendency of weak acid *ß*-lactam antibiotics in an alkaline solution will alter the dissociation pattern or dismiss certain p*K*_a_ values. This explains the poor agreement of p*K*_a_ in an alkaline environment to some degree. It also suggests the determination of an accurate p*K*_a_ value, which is not available through high pH by potentiometric titration.

Third, the micro-p*K*_a_ value of PIP is parsed among amide groups by taking AMP and AMX as references. Regularly, protonation and deprotonation of amide groups coupling with the dissociation of carboxylic group produces three probable forms in an aqueous solution for an ampholyte with weak acidity and alkalinity (Kóczián et al., 2007): cationic form [H_2_A]^+^, neutral or zwitterionic form [HA] (Martínez, 1989), and anionic form [A]^−^ (Demiralay et al., 2012). It is reported that primary amide (R-NH_2_) protonates to [R-NH_3_]^+^ when pH < 5 (Martínez, 1989; Hamada and Harris, 2006), and the acid dissociation constant of protonated R-NH_2_ is at p*K*_a_ 6–8. This pattern of primary amide agrees well with p*K*_a2_ of AMP and AMX cited in Table 2. Nevertheless, PIP does not contain primary amides.

Considering secondary amides (R_2_-NH) and tertiary amides (R_3_-N), various patterns emerge. For example, some studies report that protonated secondary amides and tertiary amides have the respective p*K*_a_ values of 10.22 and 9.45 (Cantu, Hillebrand, and Carrilho, 2005; Curtis et al., 2016). Yet, Ribeiro and Schmidt (2017) conclude that secondary amides cannot protonate (Ribeiro and Schmidt, 2017). In their work, amide groups with *ß*-lactam structures dissociate after carboxylic acid, protonated primary amide, and tertiary amide in a wide pH range. The dissociation of the protonated *ß*-lactam moiety is assigned to some p*K*_a3_ values in the range of 9.60–11.34 for AMP and AMX (Jaszczak and Kufelnicki, 2010a; Demiralay et al., 2012) (Table 2). It confirms that the secondary amide group near the *ß*-lactam structure is functionally silent to the dissociation of AMP and AMX. Additionally, many works report no p*K*_a_ value for the moiety (Table 2). It is deduced that the *ß*-lactam moiety is not responsible for the p*K*_a_ value of PIP at 8.96 ± 0.10.

Moreover, compared to AMP, PIP has an additional piperazinyl ring structure. A previous study reports that a piperazine moiety dissociates at p*K*_a_ = 9.73 and branched chain results in some shift of p*K*_a_ (Rayer et al., 2014). For example, the piperazine moiety in ciprofloxacin protonates at two protonated R_3_-N and produces two p*K*_a_ values (p*K*_a3_ = 8.70 ± 0.09 and p*K*_a4_ = 10.58 ± 0.30) (Wei et al., 2013). For PIP, a piperazinyl ring moiety suspends two carbonyl groups. Carbonyl groups may hinder the protonation ability of R_3_-N (Wuitschik et al., 2010). The piperazinyl ring of piperacillin will hydrolyze in an alkaline environment (Tsukinaka et al., 1982). Accordingly, the piperazinyl ring structure in PIP may have no chance to account for p*K*_a_ = 8.96 ± 0.10.

According to previous studies, the N-H near *ß*-lactam is stable. It cannot be oxidized by peroxymonosulfate (PMS) (Chen et al., 2018). The reaction can only take place if UV and peroxydisulfate (PDS) act together (Zhou et al., 2018). Previous studies on ampicillin (He et al., 2014), amoxicillin (Hirte et al., 2016), and cephalosporins (Qian 2014; Zhang 2015) demonstrate that N-H near *ß*-lactam is more stable than -NH_2_. Mass spectrometry studies (master dissertation in Chinese) (Chang 2018) have shown that this N-H near *ß*-lactam can also be protonated, and the N-H near the piperazine group can only be protonated if it exists alone. MS/MS studies on PIP were also conducted in this dissertation. It indicated that the N-H near the piperazine group is a chemically active site.

Thus, p*K*_a_ = 8.96 ± 0.10 of PIP is attributed to the secondary amide group (R_2_-NH) (Figure 4). As is known from mass spectrometry of PIP, direct dissociation is involved instead of protonation dissociation. Additionally, some studies report a special deprotonation phenomenon of R_2_-NHCO effected by the phenmethyl functional group (Chiu and Lo, 2000; Jaszczak and Kufelnicki, 2010b). Reactivity toward deprotonation increases due to a stereoelectronic twisting effect of the anilino group out of the plane of the benzene ring (Dombrowski et al., 2005). Some studies report the deprotonation of an amide group in peptides effected by C-termini (Chiu and Lo, 2000; Jaszczak and Kufelnicki, 2010b; Bokatzian-Johnson et al., 2012). In their work, amide nitrogens and alpha carbons of the peptide backbone must be considered alternative deprotonation sites. Thus, the characteristic spectrometry at m/z 517 and 516 indicates that 1) R_2_-NH near the piperazine structure dissociates after the carboxyl group, creating a p*K*_a_ value at 8.96 ± 0.10; 2) R_2_-NH near the *ß*-lactam structure is functionally silent to dissociation.

### 3.4 Distribution of PIP molecular forms along with pH

**FIGURE 5:**
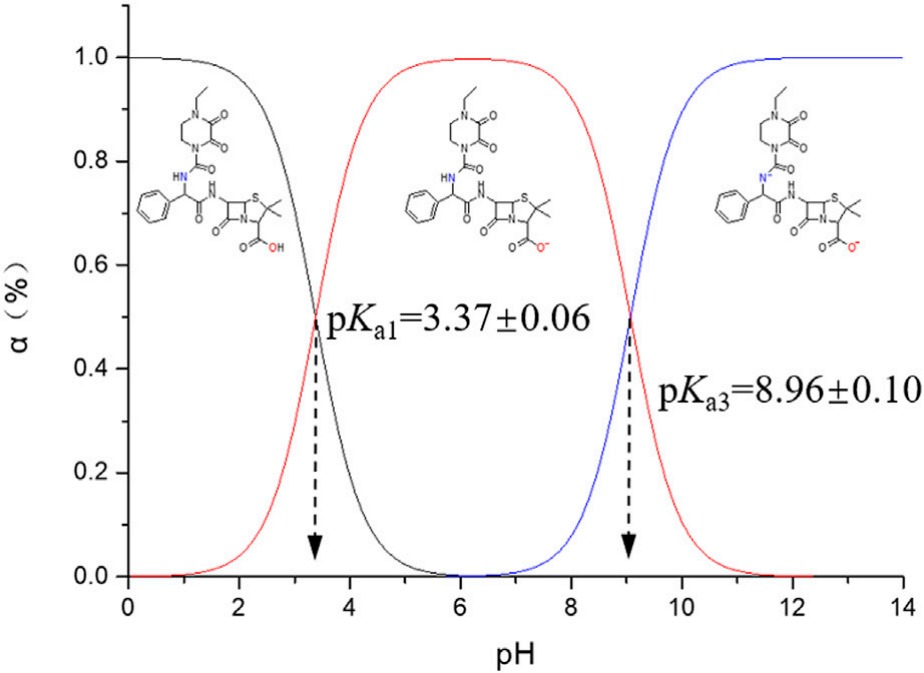
Distribution of PIP molecular forms versus pH. α (%) indicates the molar ratio of certain molecular forms.

Two p*K*_a_ values are identified for PIP in potentiometric titration from pH 3.0 to pH 11.0: p*K*_a1_ at 3.37 ± 0.06 and p*K*_a3_ at 8.96 ± 0.10, which are attributed to the carboxylic acid and secondary amide groups (R_2_-NH), respectively. Based on the aforementioned analysis about the two p*K*_a_ values, the distribution of PIP molecular forms that responds to the pH variation is elaborated in Figure 5.

Different from most *ß*-lactam antibiotics, PIP in an aqueous solution does not present any zwitterionic forms due to the absence of protonation. Additionally, PIP tends to degrade at a pH > 9, and the degradation rate accelerates along with the higher pH. Actually, a molecular form with minus two charges is rarely detected for PIP in an aqueous solution.

## 4 Conclusion

The p*K*_a_ values of PIP are determined by potentiometric titration. ESI-MS in the infusion mode (i.e., without LC) is used creatively to identify the real p*K*_a_ and mathematical p*K*_a_. Two micro-p*K*_a_ values (3.37 ± 0.06 and 8.96 ± 0.10) are recognized and attributed to carboxylic acid and secondary amide groups. The secondary amide groups near *ß*-lactam in PIP are functionally silent to dissociation. Different from other *ß*-lactam antibiotics, the pattern of direct dissociation is involved instead of the general pattern of protonation dissociation. Due to the degradation tendency of PIP in an alkaline solution environment, p*K*_a_ in a high alkaline solution is dismissed. This work suggests that p*K*_a_ values in the high pH range may not be reliable for the potentiometric titration method when hydrolytic degradation of antibiotics occurs. This work offers a reliable determination of the acid dissociation constant of PIP and a clear interpretation of the effect of stability of antibiotics on the dissociation process.

## Data Availability

The original contributions presented in the study are included in the article/Supplementary Material; further inquiries can be directed to the corresponding author.
