# Supplementary material for: Determination of the microscopic acid dissociation constant of piperacillin and identification of dissociated molecular forms
Source: Front Chem. 2023 Apr 26;11:1177128. doi: 10.3389/fchem.2023.1177128 (PMC10169600; doi:10.3389/fchem.2023.1177128)
Supplement: Supplementary file 1 [file DataSheet1.docx]

Supplementary Material

**Determination of Microscopic Acid dissociation constant of Piperacillin and Identification of Dissociated Molecular Forms**

**Guoao Li ^1^, Yaling Wang ^2,3^, Chengyi Sun ^2,3^, Fei Liu ^1^***

*** Correspondence:** Fei Liu; feiliu@cugb.edu.cn

## Supplementary Figures


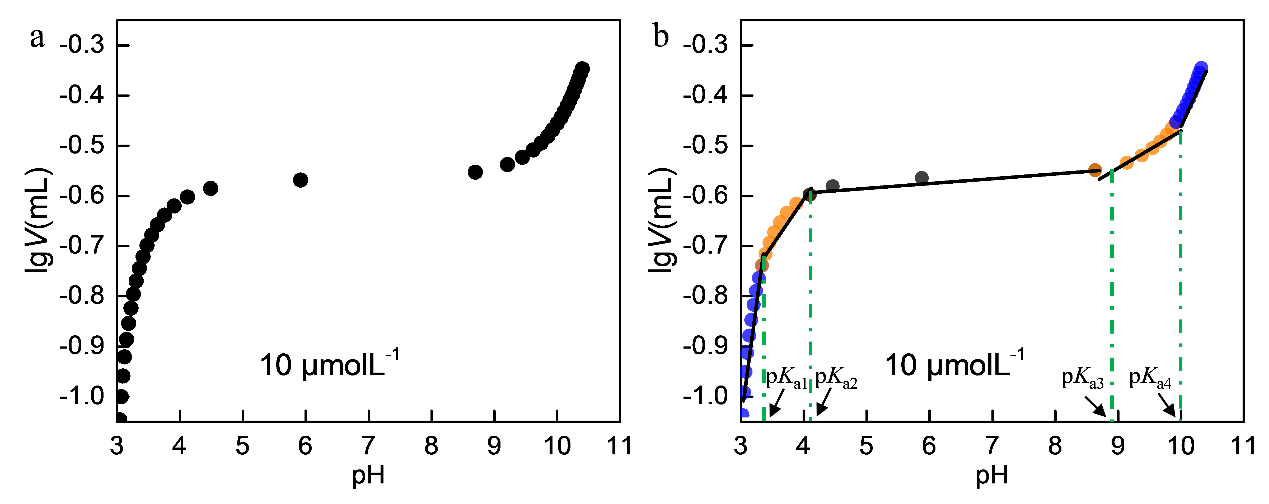


Fig. S1. Piecewise linear regression results of the lg*V*-pH data for 10 μmol L^-1^ PIP solutions. a) lg*V*-pH data; b) piecewise linear regression results.


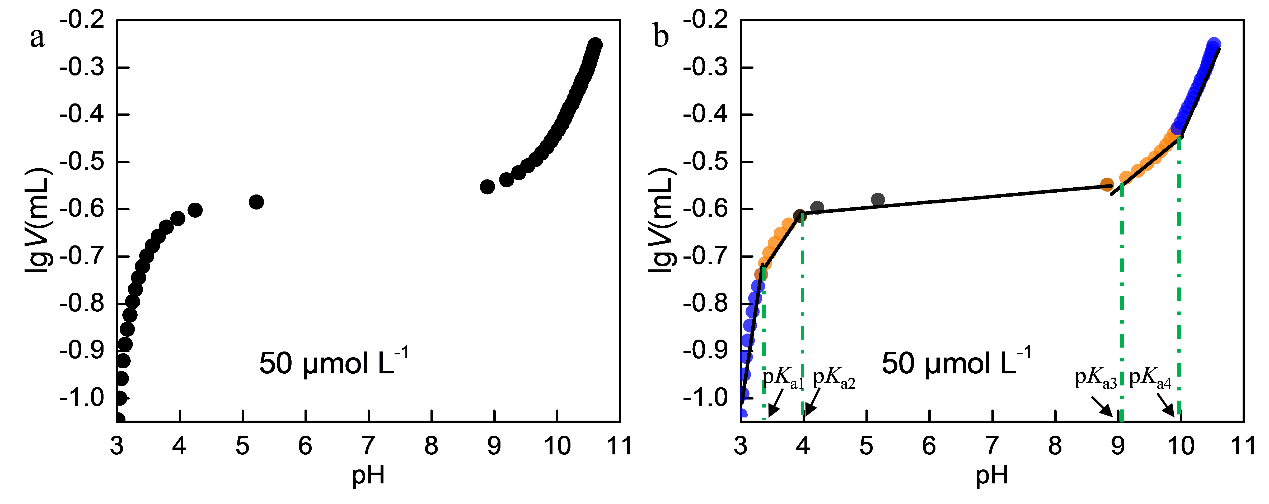


**Fig. S2.** Piecewise linear regression results of the lg*V*-pH data for 50 μmol L^-1^ PIP solutions. a) lg*V*-pH data; b) piecewise linear regression results

**2 Mass spectrometric characterization**

a

b

c

d

**m/z 259.5**

**Fig. S3**. Mass spectrometry of PIP in scanning mode (m/z 245～263). There are no multiple-charged molecular ion, for example [MH_n_]^n+^ (n=2), indicated by the absence of m/z at 259.5. This reveals that amide groups in PIP are not be protonated in acid solution.

a

b

c

d

**m/z 173**

**Fig. S4.** Mass spectrometry of PIP in scanning mode (m/z 150～184). There are no multiple-charged molecular ion, for example [MH_n_]^n+^ (n=3), indicated by the absence of m/z at 173. This reveals that amide groups in PIP are not be protonated in acid solution.

**2 Supplementary Tables**

**Table S1** Piecewise linear regression results for PIP

|  | 5 μmol L^-1^ | | 10 μmol L^-1^ | | 50 μmol L^-1^ | |
| --- | --- | --- | --- | --- | --- | --- |
| Line 1 | y=0.751x-3.283 | r^2^=0.951 | y=0.934x-3.848 | r^2^=0.950 | y=0.893x-3.702 | r^2^=0.953 |
| Line 2 | y=0.143x-1.181 | r^2^=0.910 | y=0.184x-1.341 | r^2^=0.916 | y=0.198x-1.394 | r^2^=0.936 |
| Line 3 | y=0.010x-0.624 | r^2^=0.948 | y=0.010x-0.633 | r^2^=0.815 | y=0.012x-0.657 | r^2^=0.866 |
| Line 4 | y=0.095x-1.373 | r^2^=0.961 | y=0.074x-1.209 | r^2^=0.884 | y=0.108x-1.531 | r^2^=0.937 |
| Line 5 | y=0.268x-3.113 | r^2^=0.990 | y=0.275x-3.216 | r^2^=0.990 | y=0.305x-3.498 | r^2^=0.993 |
